# Human African Trypanosomiasis Transmission, Kinshasa, Democratic Republic of Congo

**DOI:** 10.3201/eid1212.060516

**Published:** 2006-12

**Authors:** Gustave Simo, Philemon Mansinsa Diabakana, Victor Kande Betu Ku Mesu, Emile Zola Manzambi, Gaelle Ollivier, Tazoacha Asonganyi, Gerard Cuny, Pascal Grébaut

**Affiliations:** * Institute of Medical Research and Study of Medicinal Plants, Yaoundé, Cameroon;; † University of Yaoundé I, Yaoundé, Cameroon;; ‡ Programme National de Lutte contre la Trypanosomiase Humaine Africaine, Kinshasa, Democratic Republic of Congo;; § Institut National de Recherches Biomédicales, Kinshasa, Democratic Republic of Congo;; ¶ Service de Coopération et d'Action Culturelle de Kinshasa, Kinshasa, Democratic Republic of Congo;; # Institut de Recherche pour le Développement, Montpellier, France

**Keywords:** Human African trypanosomiasis. African sleeping sickness, urban transmission, Trypanosoma brucei gambiense, blood meal, Kinshasa, Democratic Republic of Congo, dispatch

## Abstract

To investigate the epidemiology of human African trypanosomiasis (sleeping sickness) in Kinshasa, Democratic Republic of Congo, 2 entomologic surveys were conducted in 2005. *Trypanosoma brucei gambiense* and human-blood meals were found in tsetse fly midguts, which suggested active disease transmission. Vector control should be used to improve human African trypanosomiasis control efforts.

Human African trypanosomiasis (HAT) (sleeping sickness) is a parasitic disease caused by a protozoan parasite belonging to the genus Trypanosoma. Approximately 60 million persons are exposed to the disease, and 500,000 are currently infected (*1*). HAT has been described as a disease affecting rural areas (*2*). During the recent increase in HAT in historic foci, emergence of foci with new epidemiologic features in urban areas was reported (*3**,**4*). Investigations of these new features showed that development of contiguous relationships between urban areas and surrounding HAT-endemic villages can create conditions favorable for HAT in urban areas (*3**–**5*). Few studies have suggested urban transmission of HAT despite potential epidemiologic consequences of such transmission (*4*).

In Kinshasa, Democratic Republic of Congo, the epidemiologic situation for HAT is complex. In 1903, Dutton-Todd reported a HAT prevalence of 2.4% in apparently healthy inhabitants of Leopoldville (*6*). In 1960, the Kinshasa focus was considered extinct, and no tsetse flies were found in the city. Until 1995, an average of 50 new cases of HAT were reported annually. However, >200 new cases have been reported annually since 1996 (e.g., 443 of 6,205 persons examined in 1998 and 912 of 42,746 persons examined in 1999) (*7*). Ebeja et al. reported that 39% of new cases were urban residents; 60% of them in the first stage of the disease (*3*).

To understand the epidemiology of HAT in this context, several investigations have been undertaken (*3**,**5**,**8*). On the basis of epidemiologic data, some investigators (*3**,**5*) have suggested that urban or periurban transmission of HAT occurs in Kinshasa. However, in a case-control study, Robays et al. concluded that HAT in urban residents of Kinshasa was linked to disease transmission in Bandundu and rural Kinshasa (*8*). To investigate the epidemiology of HAT transmission in Kinshasa, we identified and evaluated contact between humans and flies. The prevalence of Trypanosoma brucei gambiense in tsetse fly midguts was determined to identify circulation of this trypanosome between humans and tsetse flies.

## The Study

Two entomologic surveys were conducted in 2005 (during the rainy season in February and March and the dry season in June and July) at 8 sites (Ndjili Cecomaf, Ndjili Brasserie, Kimwenza, Mambre, Funa, Buma, Kimbanseke, and Kinkole) in Kinshasa. These sites were selected on the basis of HAT prevalence and entomologic data previously reported (*3*). Rural, periurban, and urban areas (Figure) were defined according to recent mapping of Kinshasa (*5*).

**Figure:**
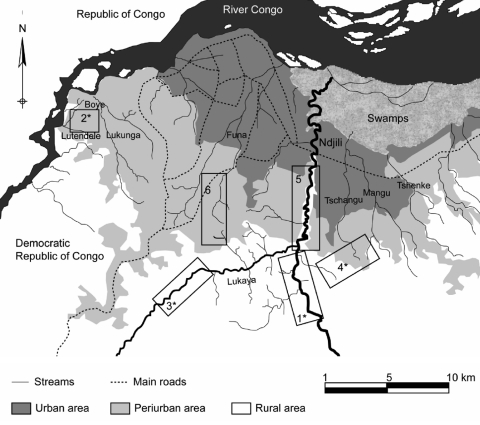
Map of Kinshasa, Democratic Republic of Congo, showing capture sites where the 2 vector surveys were conducted in urban, periurban, and rural zones. 1, Ndjili Brasseeries; 2, Mambre; 3, Kimwenza; 4, Kimbanseke; 5, Ndjili Cecomaf; 6, Funa. *Capture sites where *Trypanosoma brucei gambiense* was found in tsetse flies.

During each survey, tsetse flies were collected in pyramidal traps (*9*), and sex and species were identified. Midguts and blood meals were collected on filter paper, dried, and stored in microtubes. DNA was extracted from midguts or blood meals with 1 mL of 5% Chelex (*10*). Microtubes were incubated for 1 h at 56°C, 30 min at 100°C, and centrifuged for 10 min at 14,000 rpm. The supernatant was collected and used as DNA template for PCR. Blood meals were analyzed according to a method previously described (*11*). Trypanosomes were identified by PCR with specific primers for T. brucei s.l (*12*). and T. b. gambiense (*13*).

Because no tsetse flies were found in Buma and Kinkole during the rainy season, these 2 sites were excluded from the second survey (Figure). Entomologic data were analyzed for 610 traps from which data were obtained during the 2 surveys. A total of 897 flies of both sexes were caught; Glossina fuscipes quanzensis was the only tsetse fly species found (Table). Fresh midguts containing blood meals or trypanosomes were obtained from 570 living flies. In rural and periurban areas, 54 (9.5%, 95% confidence interval [CI] 3.7%–15.2%) teneral flies (young flies that have never taken a blood meal) were identified. Of 570 flies dissected, 117 (20.97%) had sampled blood meals and 110 were successfully identified (Table): 78 meals (67.7%, 95% CI 57.5%–75.9%) were taken from humans and 32 (27.3%, 95% CI 19%–36%) from pigs. PCR identified T. brucei s.l. in midguts of 54 (9.5%, 95% CI 7.5%– 11.5%) flies; of these, 54 flies, T. b. gambiense was found in 13. The prevalence of T. b. gambiense in tsetse midguts was 2.3% (95% CI 1.2%–3.3%). One tsetse fly with a blood meal from a pig was positive for T. b. gambiense.

**Table:** Entomologic results for tsetse flies collected during the dry and rainy seasons, Kinshasha, Democratic Republic of Congo, 2005*

Survey site	No. traps	No. flies	ADT	No. dissected flies	No. teneral flies	No. human-blood meals	No. pig-blood meals	No. *Trypanosoma brucei* s.l.	No. TBG
Ndjili Brasserie	112	423	0.94	244	25	26	18	26	9
Mambre	108	220	0.51	144	13	25	8	12	1
Kimwenza	104	139	0.33	93	6	12	3	8	2
Kimbanseke	118	57	0.12	49	6	9	2	6	1
Ndjili Cecomaf	58	35	0.15	22	3	4	1	–	–
Funa	110	23	0.05	18	1	2	–	2	–
Total	610	897	0.37	570	54	78	32	54	13

*ADT, apparent density per trap; teneral flies, young flies that have never taken a blood meal; TBG, *T. brucei gambiense* group 1.

## Conclusions

This study confirmed, as reported in previous studies, the presence of G. f. quanzensis in Kinshasa (*5*). The most favorable biotopes for tsetse flies are located along the Ndjili, Lukaya, and Boye River valleys. The apparent density per fly was low and similar to the value previously reported (*5*). Pigsties and rivers were the most favorable biotopes for tsetse flies in Kinshasa. Variations in apparent density per trap between biotopes and capture sites are probably linked to climatic factors, the environment surrounding each trap, and urbanization. Increases in human population density attract tsetse flies to rural and periurban areas and concentrate tsetse flies in regions where contact between humans and flies is possible (*14*).

Identification of tsetse flies infected with T. b. gambiense confirms contact between flies and patients. The infection rate (2.3%) in our study is comparable to the rates of 1.4% reported in Uganda and 1.9% in Brazzaville, Republic of Congo (*15*). However, T. b. gambiense midgut infection is not proof of mature infection, although it shows direct circulation of T. b. gambiense between humans and tsetse flies. Infection occurs frequently, as reflected by the feeding preference of G. f. quanzensis for humans. This finding shows that urbanization can increase transmission risk by creating conditions that may increase contact between humans and flies, as probably occurred in Kinshasa where we identified a high percentage of human-blood meals (67.7%) and tsetse flies infected with T. b. gambiense.

Our results provide evidence for local transmission of HAT in Kinshasa because we detected T. b. gambiense midgut infections, human-blood meals, and most urban resident patients in the first stage of the disease. Identification of T. b. gambiense infections in flies from different sites indicates transmission in rural and periurban areas. Some patients identified in Kinshasa could have been infected during their movement through areas outside the city for subsistence activities and economic purposes. Local transmission has likely contributed to the increase in HAT in the past decade. The sites of Ndjili Brasserie, Kimwenza, Kimbanseke, and Mambre showed higher risk for HAT transmission. We suggest that vector control be integrated into improved HAT control efforts in urban areas. In Kinshasa, focused vector control activities around pigsties and places with water-related activities can reduce fly density, contact between humans and flies, and disease transmission. Contact between tsetse flies and pigs should encourage investigations of the animal reservoir of HAT in Kinshasa. This recommendation is strengthened by the finding of T. b. gambiense in a blood meal taken from 1 pig.
